# Physical, Sexual, Emotional and Economic Intimate Partner Violence and Controlling Behaviors during Pregnancy and Postpartum among Women in Dar es Salaam, Tanzania

**DOI:** 10.1371/journal.pone.0164376

**Published:** 2016-10-18

**Authors:** Bathsheba Mahenge, Heidi Stöckl, Abdulai Abubakari, Jessie Mbwambo, Albrecht Jahn

**Affiliations:** 1 University of Dodoma, Department of Psychology, Dodoma, Tanzania; 2 Institute of Public Health, University of Heidelberg, Heidelberg, Germany; 3 Department of Global Health and Development, Faculty of Public Health and Policy, London School of Hygiene and Tropical Medicine, London, United Kingdom; 4 University for Development Studies, School of Allied Health Sciences, Community Nutrition Department, Tamale, Ghana; 5 Department of Psychiatry, Muhimbili National Hospital, Dar es Salaam, Tanzania; Royal Children's Hospital, AUSTRALIA

## Abstract

**Background:**

Intimate partner violence (IPV) during pregnancy and postpartum is a serious global health problem affecting millions of women worldwide. This study sought to determine the prevalence of different forms of IPV during pregnancy and postpartum and associated factors among women in Dar es Salaam, Tanzania.

**Methods:**

We conducted a cross-sectional study among 500 women at one to nine months postpartum in three health facilities in the three districts of Dar es Salaam: Temeke, Kinondoni and Illala. Two trained research assistants administered the questionnaire, which aimed to examine sociodemographic characteristics and different forms of IPV.

**Results:**

Of the 500 women who were interviewed, 18.8% experienced some physical and/or sexual violence during pregnancy. Forty-one women (9%) reported having experienced some physical and/or sexual violence at one to nine months postpartum. Physical and/or sexual IPV during pregnancy was associated with cohabiting (AOR 2.2, 95% CI 1.24–4.03) and having a partner who was 25 years old or younger (AOR 2.7, 95% CI 1.08–6.71). Postpartum, physical and/or sexual IPV was associated with having a partner who was 25 years old or younger (AOR 4.4, 95% CI 1.24–15.6).

**Conclusion:**

We found that IPV is more prevalent during pregnancy than during the postpartum phase. There is also continuity and maintenance of IPV during and after pregnancy. These results call for policy and interventions to be tailored for pregnant and postpartum women.

## Introduction

Intimate partner violence (IPV) has been recognized as a highly prevalent and important public health problem affecting the wellbeing and health of millions of women and children worldwide. Globally, it has been estimated that one-third of women will experience some form of physical and/or sexual violence in their lifetime and that one in three homicides of women are committed by intimate partners [1, 2].

Population-based studies across the world have found the prevalence of physical IPV during pregnancy to be between 1% and 28% [3]. A systematic review of different forms of IPV during pregnancy in antenatal care in sub-Saharan Africa found overall rates ranging from 2% to 57%: 22.5% to 40% experienced physical violence, 2.7% to 26.5% experienced sexual violence and 24.8% to 49% experienced emotional abuse [4]. In Tanzania, the prevalence of physical or sexual lifetime IPV among women was found to be 44%, with the lifetime prevalence of physical violence being 9% in Dar es Salaam alone and 12% in Mbeya [5, 6]. Further, a recent study on physical and/or sexual violence in the index pregnancy in antenatal care at a national referral hospital in Dar es Salaam found a prevalence of 27% [7].

The adverse effects of IPV during pregnancy for both mothers and their children have been well documented [8–10]. Several studies have established a stable link between IPV during pregnancy and maternal complications such as pre-term labor, miscarriages, premature rupture of membranes, abdominal pains, excessive bleeding, anemia, kidney infections, bleeding before 37 weeks, hypertension, gestational diabetes, cesarean section and poor mental health [11–14]. A study in a Tanzanian antenatal care setting found an association between IPV during pregnancy and symptoms of depression, anxiety and PTSD during pregnancy. IPV during pregnancy was associated with mothers’ younger age, being married, secondary level education and being self-employed [7]. Moreover, in a systematic review conducted in sub-Saharan Africa, IPV during pregnancy was associated with low socio-economic status, the partner’s alcohol abuse and young age [4]. IPV has also been linked to mental health effects and whole infant development during the postnatal period, and it may also affect overall infant wellbeing in terms of physical and emotional development [15].

While there is increasing evidence of the prevalence of physical and/or sexual violence in pre-and postnatal cases in sub-Saharan African countries, there is still limited data about the prevalence of different forms of pregnancy-related IPV and factors associated with IPV during and after pregnancy. This study aimed to determine the prevalence, nature and factors associated with IPV among pregnant and postpartum women in Tanzania.

## Materials and Methods

The study was conducted between December 2014 and March 2015 among women attending clinics for children under the age of 5 (also known as under-five clinics) in three health centers in Dar es Salaam. The under-five clinics promote healthy behavior and family planning, as well as offering immunizations and extra care for low birthweight babies or babies born to HIV-positive mothers. The three health centers were intentionally selected from the Temeke, Illala and Kinondoni districts of Dar es Salaam because they offered relatively similar services and had a representative number of attendees (ranging from 50 to 200 children per day). The health center in Kinondoni mainly provided antenatal care and under-five clinics, while the other two health centers offered a wider range of services, including in-patient services.

The mothers were recruited after their visits to the under-five clinic. All attending mothers aged 18 years and older who were physically fit and without cognitive impairment were invited to participate in the study. The response rate was 97%: 541 women were approached to take part in the study and only 18 declined to participate. We excluded 23 women from the study: 15 for security reasons (they attended the clinics with their partners and such partner presence could be intrusive and unsafe) and eight because they were not the biological mothers.

We used a consecutive sampling technique to select study participants for the interviews. A research assistant explained the nature of the study to each woman in a separate private room, read the consent form and obtained her verbal consent. Due to the sensitive nature and cultural context of the study, we used a verbal consent since a written consent might have implied legal consequences.

Two trained female research assistants with diplomas in nursing and midwifery administered the study questionnaires in Kiswahili, the most widely spoken language in Tanzania. The participants were not financially compensated, but refreshments were provided during the interviews. Women perceived to be in immediate danger were referred to gender-based violence intervention units, which were led by trained medical staff and social workers in the respective health centers.

The study received ethical approval from the National Institute of Medical Research (NIMR) in Tanzania and from the University of Heidelberg. It was endorsed by regional medical officers and medical officers in charge in the respective hospitals. We observed a protocol regarding the protection of human subjects which adheres to the WHO ethical guidelines [16]. Participants were assigned unique numbers to ensure confidentiality was maintained.

### Measurements

The structured questionnaire included information about the participants’ sociodemographic characteristics, such as age, education, employment status and marital status. The participants also provided information about their partner’s age, education and employment status, as well as the sex and age of the child.

Intimate partner violence was captured through the instrument from the WHO multi-country study, which includes five forms of IPV [1]. Women were asked if they experienced IPV during the index pregnancy. They were also asked if they experienced IPV after childbirth to 9 months postpartum. They answered yes or no to whether they had experienced each of the five forms of IPV: [1]

1. Physical violence (6 questions): Has your partner ever; slapped you or thrown something at you that could hurt you? Pushed you, shoved you or pulled your hair? Hit you with his fist or with something else that could hurt you? Kicked you, dragged you about or beaten you up? Attempted to choke you or burned you on purpose? Threatened to use or actually used a gun, knife or other weapon against you?
2. Sexual violence (3 questions): Has your partner ever; ever forced you to have sexual intercourse by threatening you, holding you down or hurting you in some way? Did you ever agree to have intercourse when you did not want to because you were afraid of what your husband/ partner might do if you refused? Did your partner or any other partner ever force you to do something sexual (besides vaginal intercourse) that you did not want to do?
3. Psychological violence (4 questions): Has your partner ever; insulted you or made you feel bad about yourself? Belittled or humiliated you in front of other people? Done things to scare or intimidate you on purpose (e.g., by the way he looked at you, by yelling and smashing things)? Threatened to hurt you or someone you care about?
4. Controlling behavior (5 questions): Has your partner ever; tried to keep you from seeing your friends? Tried to restrict contact with your family of birth? Insisted on knowing where you are all times? Acted jealous and get angry if you speak with another man? Often been suspicious that you are unfaithful?
5. Economic violence (two questions): Has your partner ever; taken your earnings or savings from you against your will? Refused to give you money for household expenses, even when he has money for other things?

The scale has been validated and used in Tanzania [1].

### Analysis

Data analysis was done using STATA version12. A woman was considered to have experienced IPV during and after pregnancy if she answered yes to any question within any of the five forms of IPV.

Descriptive statistics included frequencies of each variable. The first step in the analysis was to screen all potential factors that could influence the dependent variable. Variables that showed a p-value of <0.3 in the univariate models were selected and used in the logistic regression model analysis, associated factors controlled each other. A p-value <0.05 was considered to be significant and an odds ratio <1 indicates the likelihood of reduced risk, while an odds ratio >1 could be considered an increased risk.

### Results

We interviewed 500 women from the three selected under-five clinics in Dar es Salaam: 168 (33.6%) women in Kinondoni, 182 (36.4%) in Temeke and 150 (30%) in Illala. The mean age of participants was 27 years, ranging from 18 to 48 years; 116 women (23.2%) were aged 18–21 years, 332 (66.4%) were aged 22–35 years and 52 (10.4%) were aged 36 years or older. The majority of women (337; 67.4%) were married; the rest were cohabiting (84; 16.8%) or single (79; 15.8%). The lengths of their relationships ranged from 1 to 28 years. For more than half the women (278, 55.6% the child they attended the clinic with was their first child.

Primary school education or below was the most frequent level of education (375; 75%) among the participants, followed by secondary education and above (125; 25%). A large number of the participants were unemployed (292; 58.4%); the rest were self-employed (156; 31.2%) or employed (52; 10.4%).

The participants’ partners had a mean age of 32 years, ranging from 18 to 65 years. Of them, 98 (16.6%) were aged 18–25 years, 251 (50.2%) were aged 26–35 years and 151 (30.2%) were aged 36 years or older. The majority of partners (293; 58.6%) had a primary school education or below and the rest had secondary education and above. More than half of the partners were self-employed (288; 57.6%) and the rest were employed.

#### IPV during pregnancy

Of the participants, 18.8% (n = 94) reported experiencing some form of physical and/or sexual IPV during the index pregnancy, 12.4% (n = 62) reported experiencing some act of physical violence in the index pregnancy and 9% (n = 45) reported experiencing some act of sexual violence. In addition, 31% (n = 155) reported experiencing psychological violence, 15% (n = 75) reported experiencing economic violence and 48.4% (n = 242) reported experiencing controlling behavior during their index pregnancy. Some form of psychological and/or controlling behavior was experienced by 58.2% (n = 291) of the participants.

Women who were cohabiting were more likely to experience physical and/or sexual IPV in the index pregnancy compared to their single counterparts (AOR 2.2, 95% CI 1.24–4.03). If they had partners aged 25 years or younger, they were more likely to experience physical and/or sexual IPV during their index pregnancy than women with partners aged 26–35 years (AOR 2.7, 95% CI 1.08–6.71). Other factors were not significant (Table 1).

**Table 1:** The sociodemographic characteristics and physical and/or sexual IPV experienced by the participants during the index pregnancy (n = 500).

	Physical and/or sexual violence IPV during pregnancy					
	Yes	No	OR	CI	AOR	CI
**Participant’s age**						
≥36	8 (15.4%)	44 (84.6%)	ref			
≤21	33 (28.5%)	83 (71.6%)	2.1	.93–5.13	.78	.24–2.51
22–35	52 (15.7%)	280 (84.3%)	1.0	.45–2.29	.58	.21–1.62
**Marital status**						
Married	48 (14.2%)	289 (85.8%)	ref			
Single	21 (26.6%)	58 (73.4%)	2.1	1.21–3.91**	1.7	.96–3.37
Cohabiting	24 (28.6%)	60 (71.4%)	2.4	1.4–4.23**	2.2	1.24–4.03**
**Participant’s education**						
Secondary and above	18 (14.4%)	107 (85.6%)	ref			
Primary and below	75 (20%)	300 (80%)	1.5	.84–2.6	1.4	.78–2.69
**Participant’s employment**						
Employed	6 (11.5%)	46 (88.5%)	ref			
Unemployed	65 (22.3%)	227 (77.7%)	.46	.18–1.11	1.8	.75–4.82
Self-employed	22 (14.1%)	134 (85.9%)	.79	.30–2.08	1.2	.46–3.37
**Partner’s age**						
≥36	17 (11.3%)	134 (88.7%)	ref			
≤25	28 (28.6%)	70 (71.4%)	3.2	1.61–6.15***	2.7	1.08–6.71*
26–35	48 (19.1%)	203 (80.9%)	1.9	1.02–3.38*	2.1	.98–4.56
**Partner’s education**						
Secondary and above	35 (16.9%)	172 (83.1%)	ref			
Primary and below	58 (19.8%)	235 (80.2%)	.82	.52–1.31	1.2	.76–2.10

“*** = p<0.001”

“** = p<0.005”

“* = p<0.05” Associated factors control for each other.

Married women were more likely to experience psychological violence in the index pregnancy than their single counterparts (AOR 1.8, 95% CI 1.108–3.09). Women who were cohabiting (AOR 2.4, 95% CI 1.16–4.96), single (AOR 8.2 CI 4.2–15.8) or who had a partner with a primary school education (AOR 2.2, 95% CI 1.18–4.09) were more likely to experience economic violence.

There was also an overlap between different forms of IPV during pregnancy (S1 Fig).

#### IPV postpartum

Some participants reported IPV between one and nine months postpartum. 8.2% (n = 41) reported physical and/or sexual violence, 5.2% (n = 26) reported physical violence and 3.8% (n = 19) reported sexual violence. Psychological violence postpartum was reported by 17.8% (n = 89) of participants, economic violence by 11.4% (n = 57) and controlling behavior by 44.4% (n = 222).

In addition, some participants only experienced violence postpartum and not during pregnancy. Of all the participants, 5% (n = 25) experienced psychological IPV, 2.6% (n = 13) experienced physical IPV, 1.6% (n = 8) experienced sexual IPV, 2.6% (n = 13) experienced economic IPV and 4.6% (n = 23) experienced controlling behaviors.

Women with partners aged 25 years or younger were more likely to experience physical and/or sexual violence postpartum (AOR 4.4, 95% CI 1.24–15.6; Table 2). Women aged 36 years or older were more likely to experience controlling behaviors postpartum (AOR 2.2, 95% CI 1.06–4.46). Single women (AOR 4.3, 95% CI 1.7–10.2) and those who had a partner aged 25 years or younger (AOR 3.2, 95% CI 1.41–7.31) were more likely to experience economic violence postpartum.

**Table 2:** The participants’ sociodemographic characteristics and experiences of physical and/or sexual IPV at one to nine months postpartum (n = 500).

	Physical and/or sexual violence IPV after pregnancy					
	Yes	No	OR	CI	AOR	CI
**Participant’s age**						
≥36	1 (1.9%)	51 (98.1%)	ref			
≤21	11 (9.5%)	105 (90.5%)	5.3	.67–5.21	1.7	.16–17.8
22–35	29 (8.7%)	303 (91.3%)	4.8	.65–3.9	2.1	.23–18.9
**Marital status**						
Married	27 (8.0%)	310 (92.0%)	ref			
Single	5 (6.3%)	74 (93.7%)	0.7	.28–2.08	0.6	.21–1.67
Cohabiting	9 (10.7%)	75 (89.3)	1.3	.62–3.05	1.16	.51–2.63
**Participant’s education**						
Secondary education and above	10 (8.0%)	115 (92.0%)	ref			
Primary education and below	31 (8.3%)	344 (91.7%)	1.03	.49–2.17	1.3	.59–3.06
**Employment**						
Employed	5 (9.2%)	47 (90.8%)	ref			
Unemployed	23 (7.9%)	269 (92.1%)	1.24	.45–3.43	0.72	.25–2.11
Self-employed	13 (8.3%)	143 (91.7%)	1.17	.39–3.45	0.88	.28–2.72
**Partner’s age**						
≥36	5 (3.3%)	146 (96.7%)	ref			
≤25	12 (12.2%)	86 (87.8%)	4.1	1.3–11.9*	4.4	1.24–15.6*
26–35	24 (9.6%)	227 (90.4%)	3.1	1.2–8.2*	2.7	.92–8.10
**Partner’s education**						
Secondary and above	19 (9.2%)	188 (90.8%)	ref			
Primary and below	22 (7.5%)	271 (%)	1.24	.66–2.36	0.79	.39–1.59

“* = p<0.05” Associated factors control for each other.

There was also a trend of overlap between different forms of IPV postpartum (S2 Fig). Women also experienced more than one form of IPV postpartum.

#### The overlap of IPV during pregnancy and postpartum

Of the 500 participants, 4.8% (n = 24) reported physical and/or sexual IPV and 12.6% (n = 63) experienced psychological IPV and/or controlling behaviors both during pregnancy and postpartum. 13.8% (n = 69) of the participants reported experiencing physical and/or sexual IPV only during pregnancy, and 3.4% (n = 17) of them experienced physical and/or sexual IPV for the first time postpartum.

Thus, 18.6% (n = 93) of the participants experienced postpartum physical and/or sexual IPV, which was a continuation of their pre-delivery experience. However, postpartum IPV was a new experience for a few women (3.4%; n = 17). There were marked differences between the victims and perpetrators in both sub-groups in terms of age, marital status and partner age. Women aged 21 years and younger experienced more physical and sexual IPV during pregnancy only (22.4%; n = 26) than their counterparts, while those between the ages of 22 and 35 years experienced new physical and/or sexual IPV postpartum (3.6%; n = 12). Single women experienced more physical and/or sexual IPV during pregnancy alone (21.5%; n = 17), while married women experienced more physical and/or sexual IPV postpartum (4.2%; n = 14). Men who were under the age of 25 years were less likely than their counterparts to be the perpetrators of physical and/or sexual IPV during pregnancy (43%; n = 21) and postpartum (5.1%; n = 5).

## Discussion

This study found IPV during pregnancy to be more prevalent than IPV at one to nine months postpartum. It further found a 18.6% rate of physical and/or sexual violence during the index pregnancy and an 8% frequency postpartum. Controlling behaviors were the most prevalent form of IPV both during pregnancy (48.4%) and at one to nine months postpartum (44%), followed by psychological IPV (31% and 17.8%, respectively).

These rates are within the 2% to 57% range of different forms of IPV during pregnancy reported in a systematic review of African countries [4]. However, the rate of physical and/or sexual violence during the index pregnancy found in this study was lower than that found in a previous study conducted in Tanzania (which found a prevalence of 27%) [17]. Potential explanations for this disparity are the type and settings of the clinics, as the previous study was done in a tertiary care clinic. The discrepancy could also be due to using a different tool: the previous study used the Conflict Tactics Scale, which might have allowed for inclusion of less severe forms of violence [18].

To our best knowledge, this is the first study to investigate psychological and economic abuse and controlling behavior during and after pregnancy in Tanzania. The high rates of controlling behavior and psychological and economic abuse are of great concern, as a study conducted among pregnant women in the USA found a strong link between psychological IPV and posttraumatic stress disorder [19]. Additionally, an investigation among Philippine women linked both economic and psychological abuse to suicidal incidents and psychological distress [20]. Ludermir highlighted the need to seriously consider psychological IPV in interventions, noting that most of them currently emphasize physical IPV [21].

The decline of IPV after one to nine months postpartum found in this study is consistent with some other studies which also reported a decline of IPV during the postpartum phase [22]. However, a study conducted in three provinces in China found that abuse was reported to have increased among women after 11 months during the postpartum phase [23]. Despite the lower rates of IPV after pregnancy, other studies have also found a continuity and maintenance of violence [22, 24]. Postpartum IPV is said to be common among unemployed women, those whose partners have abused alcohol, those who have been in their relationship for less than one year and those with a very controlling partner [25].

This study found that women are still likely to suffer postpartum IPV, even if they did not experience IPV during pregnancy. Although the postpartum IPV percentages might be smaller than those of IPV during pregnancy, such acts of violence still call for attention due to the potential adverse effects of IPV in the postpartum phase [26]. There is a need for policy and screening geared toward identifying new cases. IPV not only affects the mother; infants and children are also at risk of developing mental health and behavioral problems. Intergenerational transmission of violence also remains a threat, as there is a link between IPV and child abuse [27].

In the univariate analysis higher odds for women with partners aged 25 or younger was observed. This conforms to the findings of other studies. For example, IPV during pregnancy was associated with mothers’ younger age in Tanzania [7]. Moreover, in a systematic review conducted in sub-Saharan Africa, IPV during pregnancy was associated with young age [4]. However, there was a reverse association observed in the mutual adjusted model between partner age and IPV occurrence. Perhaps this observation was due to the interaction between partner age, maternal age and partner’s education.

This study has several limitations which have to be taken into account. First, it was a cross-sectional study and thus cannot establish a causal link between IPV during the pregnancy and postpartum. Second, since the study was conducted in urban health facilities in Dar es Salaam, the results cannot be extrapolated to represent the situation in the entire country. Additionally, due to the sensitive nature of the study in terms of social stigma and IPV being seen as a private matter, the participants may have underreported IPV. The exclusion of women accompanied by their partners may have resulted in an underrepresentation of severe cases of IPV, especially controlling behaviors.

Although the study was cross-sectional, meaning recall bias was inevitable, participants were given clues to assist them in remembering what they went through during and after pregnancy. We also took steps to ensure we established rapport with the women so they could speak freely.

### Conclusion

The findings reveal that IPV is prevalent and the rates of controlling behavior are high both during pregnancy and postpartum. As highlighted in the results, maintenance and continuity of IPV persisted during pregnancy and postpartum. Some new cases of IPV emerged during the postpartum phase, but some IPV also stopped after pregnancy. These findings show that women can suffer IPV in these two phases of their life, which could lead to severe psychological and physical health implications. These results call for policy and interventions to be tailored to pregnant and postpartum women.

## Supporting Information

S1 Fig (TIF)

S2 Fig (TIF)

S1 File (PDF)
