# Supplementary figures and images for: Physical, Sexual, Emotional and Economic Intimate Partner Violence and Controlling Behaviors during Pregnancy and Postpartum among Women in Dar es Salaam, Tanzania
Source: PLoS One. 2016 Oct 18;11(10):e0164376. doi: 10.1371/journal.pone.0164376 (PMC5068783; doi:10.1371/journal.pone.0164376)

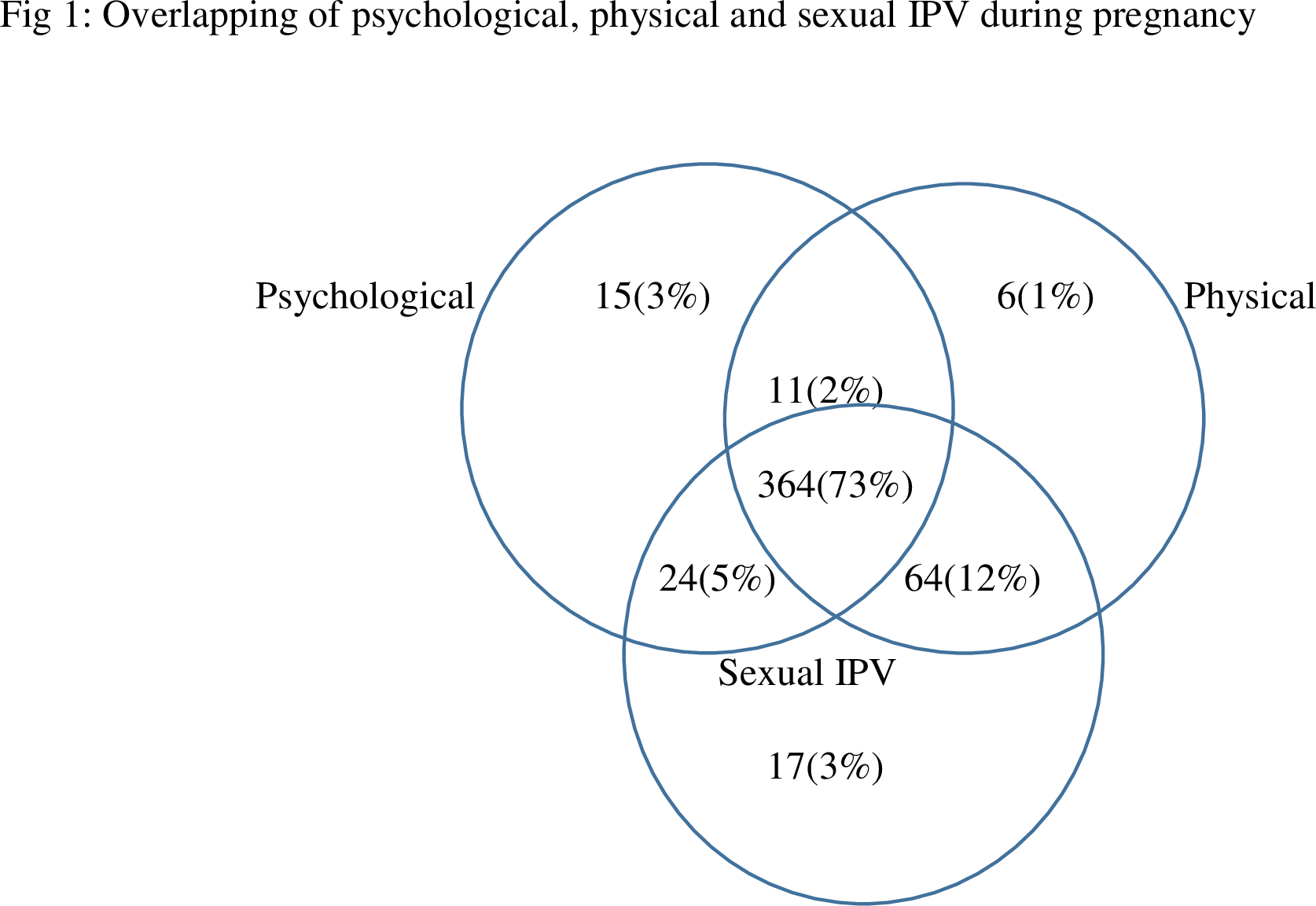

Supplement: S1 Fig — (TIF) [file pone.0164376.s001.tif]

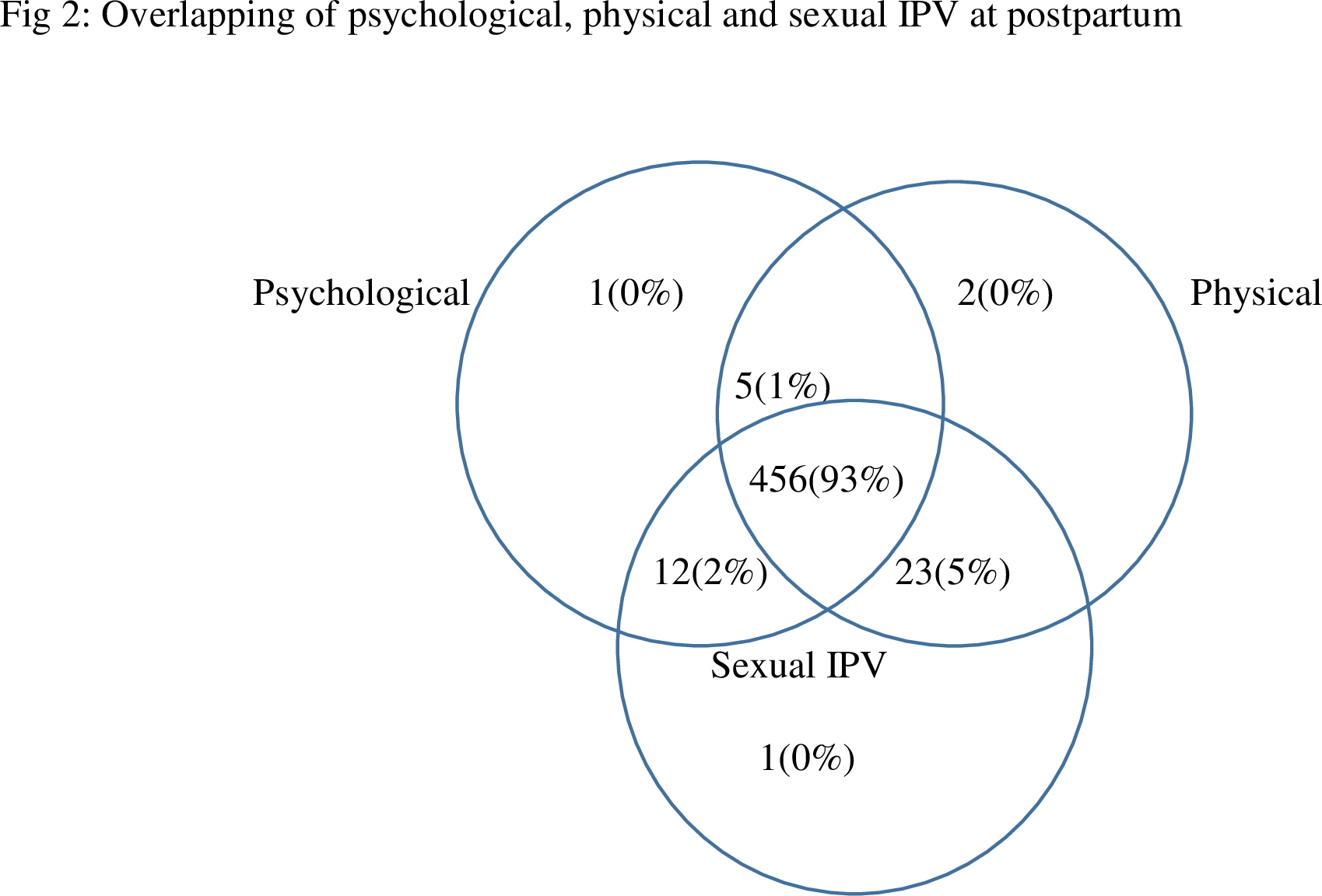

Supplement: S2 Fig — (TIF) [file pone.0164376.s002.tif]
