# Supplementary material for: Physical, Sexual, Emotional and Economic Intimate Partner Violence and Controlling Behaviors during Pregnancy and Postpartum among Women in Dar es Salaam, Tanzania
Source: PLoS One. 2016 Oct 18;11(10):e0164376. doi: 10.1371/journal.pone.0164376 (PMC5068783; doi:10.1371/journal.pone.0164376)
Supplement: S1 File — (PDF) [file pone.0164376.s003.pdf]

General Information

Date of interview: ....

Name of interviewer: ....

II. DEMOGRAPHIC CHARACTERISTICS OF RESPONDENT

Number of interviewee:..

1. What is your date of birth?
2. What is you currently marital status?
    a. Married monogamous
    b. Married polygamous
    c. cohabiting
    d. Single
    e. Separated
    f. Divorced
    g. Widowed
3. How long have you been together with your partner?.......
4. How many children have you given birth to? _____
5. What is your education level
    a. No formal education
    b. Primary education
    c. Secondary Education
    d. College/ University
6. What is your employment status?
    a. Employed
    b. Unemployed
    c. Self employed

Now I would like to ask you a little more about your main current partner/husband.

7. How old is he?_____ years
8. Primary partner/Husband education level
    a. No formal education
    b. Primary education

c. Secondary education

d. College/university education

9. What is his employment status?

a. Employed

b. Unemployed

c. Self employed

10. Does your household have?

a. Electricity?
b. a paraffin lamp
c. a radio
d. a television
e. a mobile telephone
f. an iron (charcoal or electric)
g. a refrigerator

11. What type of fuel does your household mainly use for cooking?
a. electricity
b. bottled gas
c. paraffin / kerosene
d. charcoal
e. firewood
f. crop residuals, straw, grass 06
g. animal dung

13. What is the main source of energy for lighting in the household?
a. electricity
b. solar
c. gas
d. paraffin-hurricane lamp
e. firewood
f. candles

14. Does any member of your household own:
a. a bicycle
b. motorcycle/scooter
c. car/truck

I would now like to ask you a few questions on your pregnancy

15. When you got pregnant, did you want to get pregnant at that time?

a. Yes

b. No

17. Have you ever had a pregnancy that miscarried, was aborted or ended in still birth?

a. Yes

b. No

18. Have you ever given birth to a boy or girl who was born alive and later died?

a. Yes

b. No

Ask for the antenatal card and tick if any of the mentioned complication were recorded (if she does not have it, record her phone number at least two phone numbers…………………………………………….)

	**Maternal complication**	**Yes**	**No**
1	Pre eclampsia		
2	Gestational diabetes		
3	Abdominal pain		
4	Bleeding before 37 weeks		
5	Urinary tract infection		
6	Premature rupture of membranes		
7	Diminished fetal movements		
8	False labour		
9	Growth retardation		
10	Spontaneous premature labour		
11	backache		
12	headache		
13	Caesarean section		
14	STIs		

Infant's weight at birth ………….. Kgs

Apgar score ……… at one minute …………at 5 minutes

Current age of the child………………….months

Has the baby had any of the following in the last two weeks?

		Yes	No
1	Diarrhea		

2	Fever		
3	Cough or difficulty in breathing		
4			
5			

Postpartum depression

The Hopkins Symptom Checklist

The following are symptoms which might be bothering in the past days, highlight symptoms according to their extremes

**No**		**1=Not at all**	**2=A little**	**3=Quite a bit**	**4=Extremely**
1	Being suddenly scared for no apparent reason				
2	Feeling fearful				
3	Feeling fearful, faintness, dizziness, or weakness				
4	Nervousness or shakiness inside				
5	Heart pounding or racing				
6	Trembling				
7	Feeling tense or keyed up				
8	Headaches				
9	Spells of terror or panic				
10	Feeling restless, not being able to sit still				
11	Feeling low in energy, slowed down				
12	Blaming oneself for things				
13	Crying easily				
14	Loss of sexual interest or staying asleep				
15	Poor appetite				
16	Difficulty falling asleep or staying asleep				

17	Feeling hopeless about the future				
18	Feeling blue				
19	Feeling lonely				
20	Thoughts of ending one's life				
21	Feeling trapped or caught				
22	Worrying too much about things				
23	Feeling no interest on things				
24	Feeling everything is an effort				
25	Feelings of worthlessness				

Childhood Adverse Experiences Questionnaire

These next questions are about certain things YOU may have experienced. When you were growing up, during the first 18 years of your life ….						
		Many Times	A few Times	Once	Never	refused
1	Did a parent, guardian or other household member yell, scream or swear at you, insult or humiliate you?					
2	Did a parent, guardian or other household member threaten to, or actually, abandon you or throw you out of the house?					
3	Did a parent, guardian or other household member spank, slap, kick, punch or beat you up?					
4	Did a parent, guardian or other household member hit or cut you with an object, such as a stick (or cane), bottle, club, knife, whip etc					

5	Did someone touch or fondle you in a sexual way when you did not want them to?					
6	Did someone make you touch their body in a sexual way when you did not want them to?					
7	Did someone attempt oral, anal, or vaginal intercourse with you when you did not want them to?					
8	Did someone actually have oral, anal, or vaginal intercourse with you when you did not want them to?					

Intimate Partner Violence

Section A: During Pregnancy

The next questions are about things that happen to many women, and that your current partner, or any other partner may have done to you

Psychological abuse questions

1. Did your husband/partner or any other partner (previous partners)do any of these during your last pregnancy?

	Acts of violence	Yes	No
a	Insulted you or made you feel bad about yourself?		
b	Belittled or humiliated you in front of other people?		
c	Done things to scare or intimidate you on purpose (e.g. by the way he looked at you, by yelling and smashing things)?		
d	Threatened to hurt you or someone you care about?		

Physical Abuse

2. Has your husband or any other partner ever done to you, any of this during your last pregnancy?

		Yes	No
a	Slapped you or thrown something at you that could hurt you		
b	Pushed you or shoved you or pulled your hair?		
c	Hit you with his fist or with something else that could hurt you?		

d	Kicked you, dragged you about or beaten you up?		
e	Attempted to choke you or burned you on purpose?		
f	Threatened to use or actually used a gun , knife or other weapon against you?		

Sexual Abuse

3. During your last pregnancy did your current, partner or any other

		Yes	No
a	Ever force you to have sexual intercourse by threatening you, holding you down or hurting you in some way?		
b	Did you ever agree to have intercourse when you did not want to because you were afraid of what your husband/ partner might do if you refused?		
c	Did your partner or any other partner ever force you to do something sexual (besides vaginal intercourse) that you did not want to do?		

Controlling behavior

4. During your last pregnancy did your husband, current partner or any other do any of the following?

		Yes	No
a	Try to keep you from seeing your friends?		
b	Try to restrict contact with your family of birth?		
c	Insist on knowing where you are all times?		
d	Is jealous and gets angry if you speak with another man?		
e	Is often suspicious that you are unfaithful?		

Economic abuse

5. During your last pregnancy did your husband /partner or any other partner

		never	Once or twice	Several times	Many times/all the times	Does not have savings or earnings	Do not remember	Refused no answer
a	Taken your earnings or savings from you against your will?							
b	Refuse to give you money for household expenses, even when he has money for other things?							

Section B: Postpartum

Psychological abuse

1. After your last pregnancy Did your husband/partner or any other partner do any of these?

	Acts of violence	Yes	No
a	Insulted you or made you feel bad about yourself?		
b	Belittled or humiliated you in front of other people?		
c	Done things to scare or intimidate you on purpose (e.g. by the way he looked at you, by yelling and smashing things)?		
d	Threatened to hurt you or someone you care about?		

Physical Abuse

2. After your last pregnancy Has your husband or any other partners ever done to you, any of this?

		Yes	No
a	Slapped you or thrown something at you that could hurt you		
b	Pushed you or shoved you or pulled your hair?		
c	Hit you with his fist or with something else that could hurt you?		
d	Kicked you, dragged you or beaten you up?		
e	Choked you or burned you on purpose?		
f	Threatened to use or actually used a gun , knife or other weapon against you?		

Sexual Abuse

3. After your last pregnancy did your current or previous partner after your last pregnancy

		Yes	No
a	Ever force you to have sexual intercourse by threatening you, holding you down or hurting you in some way?		
b	Did you ever agree to have intercourse when you did not want to because you were afraid of what your husband/ partner might do if you refused?		
c	Did your partner or any other partner ever force you to do something sexual (besides vaginal intercourse) that you did not want to do?		

Controlling behavior

4. After your last pregnancy did your husband, current partner or any other do any of the following?

		Yes	No
a	Try to keep you from seeing your friends?		
b	Try to restrict contact with your family of birth?		
c	Insist on knowing where you are all times?		
d	Is jealous and gets angry if you speak with another man?		
e	Is often suspicious that you are unfaithful?		

Economic abuse

5. After your last pregnancy did your husband /partner or any other partner

		never	Once or	Several times	Many times/all	Does not have	Do not remember	Refused no answer

			twice		the times	savings or earnings		
a	Taken your earnings or savings from you against your will?							
b	Refuse to give you money for household expenses, even when he has money for other things?							
